# Nanoclay-Reinforced Glass-Ionomer Cements: In Vitro Wear Evaluation and Comparison by Two Wear-Test Methods

**DOI:** 10.3390/dj5040028

**Published:** 2017-10-19

**Authors:** Muhammad A. Fareed, Artemis Stamboulis

**Affiliations:** 1 Adult Restorative Dentistry, Biomaterials and Prosthodontics Oman Dental College, Muscat 116, Sultanate of Oman; 2 School of Metallurgy and Materials, University of Birmingham, Edgbaston, Birmingham B15 2TT, UK; a.stamboulis@bham.ac.uk

**Keywords:** glass ionomer cement, oscillating wear, OHSU wear simulator, nanoclays, hardness

## Abstract

Glass ionomer cement (GIC) represents a major transformation in restorative dentistry. Wear of dental restoratives is a common phenomenon and the determination of the wear resistance of direct-restorative materials is a challenging task. The aim of this paper was to evaluate the wear resistance of novel glass ionomer cement by two wear-test methods and to compare the two wear methods.The wear resistance of a conventional glass ionomer cement (HiFi Advanced Health Care Kent, UK) and cements modified by including various percentages of nanoclays (1, 2 and 4 wt %) was measured by a reciprocating wear test (ball-on-flat) and Oregon Health and Sciences University’s (OHSU) wear simulator. The OHSU wear simulation subjected the cement specimens to three wear mechanisms, namely abrasion, three-body abrasion and attrition using a steatite antagonist. The abrasion wear resulted in material loss from GIC specimen as the steatite antagonist forced through the exposed glass particles when it travelled along the sliding path.The hardness of specimens was measured by the Vickers hardness test. The results of reciprocation wear test showed that HiFi-1 resulted in the lowest wear volume 4.90 (0.60) mm^3^ (*p* < 0.05), but there was no significant difference (*p* > 0.05) in the wear volume in comparison to HiFi, HiFi-2 and HiFi-4. Similarly, the results of OHSU wear simulator showed that the total wear volume of HiFi-4 1.49 (0.24) was higher than HiFi-1 and HiFi-2. However, no significant difference (*p* > 0.05) was found in the OHSU total wear volume in GICs after nanoclay incorporation. The Vickers hardness (HV) of the nanoclay-reinforced cements was measured between 62 and 89 HV. Nanoclay addition at a higher concentration (4%) resulted in higher wear volume and wear depth. The total wear volumes were less dependent upon abrasion volume and attrition volume. The total wear depths were strongly influenced by attrition depth and to some extent by abrasion depth. The addition of nanoclay in higher wt % to HiFi did not result in significant improvement in wear resistance and hardness. Nonetheless, wear is a very complex phenomenon because it is sensitive to a wide number of factors that do not necessarily act in the same way when compared using different parameters.

## 1. Introduction

In the development of dental materials, the main aim is to find a biocompatible and long-lasting material that can bind to the tooth structure permanently and have desirable therapeutic effects [1]. Glass ionomer cement (GIC) has evolved during the past 45 years into diverse dental products used as direct restoratives, luting agents, liner and bases, pit and fissure sealants, atraumatic and minimum-invasive materials, and as endodontic sealers. GIC is formed by an acid–base reaction between the polyacid liquid (acid) and the fluoro-alumino-silicate glass powder (base). In the setting reaction of GIC, the glass structure is attacked by acid that results in glass hydrolysis and the consequent release of Ca^2+^ and Al^3+^ cations and F^−^, PO_4_^3−^ anions. The metal cations are subsequently chelated by the carboxylate groups and crosslinking of the polyacid chains takes place to form the cement matrix [1,2]. GIC is a preferred choice of clinicians in the non-stress-bearing build-up, sandwich restoration, tunnel restoration, root caries and long-term provisional restorations. Despite several advantages of GIC such as fluoride release [2], physicochemical bonding to enamel and dentine, similar coefficient thermal expansion as of the natural tooth and the facilitation in remineralisation of caries-affected dentine [3], the main disadvantage is their low wear resistance in sites subjected to higher occlusal forces and lack of sufficient fracture toughness [3,4].

Wear is a complex tribological event that results in the loss of material due to the interfacial contact of the two surfaces. In the oral cavity, wear of dental restoratives occurs when opposing teeth come in contact and a load is applied during mastication or other functional or parafunctional activities [5]. Several test methods are reported in the literature for wear evaluation of dental restorative materials. For example, in vitro wear was determined by pin-on-disc or ball-on-flat methods [6] and by a variety of human oral simulator devices which simulate oral wear mechanism [7]. According to the American Society of Materials (ASM), there are a range of parameters that influence the wear mechanisms [8], which include the material itself, the shape and contour of the antagonist, the surface roughness, the motion and frequency of motion, the loading rate, the lubrication and the local environment (Table 1). The human oral environment is very dynamic and the fundamental wear mechanisms operating in the oral environment include abrasion, three-body abrasion, attrition, adhesion, fatigue and erosion wear or any combination of these interactions [9,10]. Furthermore, the classification of in vitro wear determination is either based on the type of movement (reciprocating, rolling, impact oscillation and flow) or on the mechanism of wear [7,8,9,10]. The aim of the present study was to compare two wear test methods and to investigate the effects of nanoclay reinforcement on the wear and microhardness of GICs. The incorporation of small amounts of nanoclay as reinforcing fillers in dental materials has shown a remarkable impact on mechanical properties [11]. The nanoclay (montmorillonite) used in this work is a member of the smectite clay family (2:1 layered silicates or phyllosilicates), and has a larger adsorption capacity due to the unique sheet-type or plate-like structure (thickness ≈ 1 nm, the width and length may differ).For this purpose, the liquid portion of GIC was modified by the dispersion of medical grade nanoclay [12] to determine the wear characteristics and Vickers hardness of nanoclay-reinforced glass ionomer cements.

## 2. Materials and Methods

### 2.1. Materials

HiFi glass powder (alumino-silicate glass) and HiFi polyacrylic acid (PAA) powder (M_w_~60,000) of commercial grade were obtained from Advanced Healthcare Limited (Kent, UK). Medical-grade nanoclay was supplied by NRC Nordmann, Rassmann GmbH (Hamburg, Germany). The nanoclay has a plate like structure with a two-to-one layered smectite clay mineral having an alumina octahedral sheet sandwiched between two tetrahedral sheets of silica (1 nm thickness and 300–600 nm surface dimensions) [12].

### 2.2. Sample Preparation

Initially, 0.10 g, 0.20 g and 0.40 g (1.0, 2.0 and 4.0 wt %) nanoclays were stirred in deionized water at 75 °C on a hot plate (Stable Temp Cole-Parmer, IL, USA) for 2 h using a magnetic stirrer at 100 rpm. The polymer solutions (liquid portion of GIC) were prepared by adding 4.0 g HiFi PAA powder (M_w_~60,000) in the above-mentioned deionized aqueous suspension containing nanoclays and stirred for 22 h. The polymer solutions were labeled as PAA, PAA1, PAA2 and PAA4. GIC specimens for wear and hardness tests were prepared by hand mixing the HiFi glass powder with the corresponding polymer liquid according to the manufacturer’s instructions. The schematic presentation of samples prepared in this study is given in Table 2.

### 2.3. Wear Test

The wear studies were performed by a reciprocating wear test and the OHSU wear simulator.

#### 2.3.1. Reciprocating Wear Test

A reciprocating tribometer was used to determine the wear resistance of cements in accordance with the American Society for Testing and Materials (ASTM) standard G133-05 [13]. The alumina ball (Spheric Trafalger Limited Sussex, UK) of 12.5 mm of diameter and average surface roughness (Ra) of 0.01 μm was used as an antagonist. The alumina ball was tightly mounted in a stainless steel holder to prevent slippage during the test. The rectangular-shaped cement specimens (10 mm of length, 5 mm of width, 2 mm of thickness) were prepared using a split brass mold and were stored in a distilled water bath at 37 °C for 22 h. After 23 h of the start of mixing, the GIC samples were removed from water and the top surfaces were wet-ground with an 800- and 1200-grit silicon carbide paper and then they were attached to a custom-made (10 mm of diameter) aluminum disc to prevent slippage or buckle during the test. A 20-N load (equivalent to the light biting force of human) [14] was applied to have a contact between the alumina ball and the flat cement specimen. The test was conducted in distilled water at room temperature with a sliding stroke length of 6 mm, a frequency of 1 Hz. The number of cycles was 10,000. The following equations were used according to the above mentioned ASTM standard to compute the sliding distance or number of cycles [13]:

(1) X=0.002×t×f×L

(2) N=t×f

where, *X* is the sliding distance of ball in meters, *N* is the number of cycles of the test, *t* is time in seconds, *f* is the oscillating frequency in Hz (cycles/s) and *L* is the length of stroke in mm.

**Figure 1:**
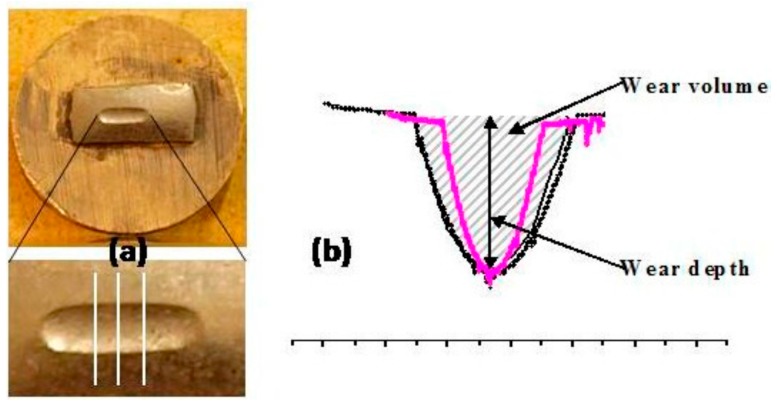
Wear facet produced by the reciprocating wear test method. The image was taken after gold coating of the mounted sample. If the material and environmental factors remain constant, the volume loss is linear with time, but the relationship of the wear volume and wear depth is not necessarily linear. (**a**) The white lines represent three estimated positions of the scans measured by the stylus profilometer and, (**b**) wear profiles from the reciprocation wear test showing same wear depth (purple line determined by the stylus profilometer), but different volume.

Five rectangular shape specimens of each cement group were tested. The area across the wear scar profile was measured by a surface roughness measuring stylus profilometer (Surf-corder, Mitutoyo, UK). The wear area was calculated using Microcal Origin 6.0 analytical software (OriginLab Corporation, Northampton, MA, USA) by integrating the area across the wear scar profile measured by the stylus profilometer and multiplying by the circumference length of the track. The wear volume was determined by multiplying the wear area and length of the wear track, which is expressed as volume loss per unit sliding distance per unit contact load. The post-wear examination of the wear track was conducted using an optical microscope (Figure 1a).

#### 2.3.2. Oregon Health and Science University (OHSU) Wear Simulator

**Figure 2:**
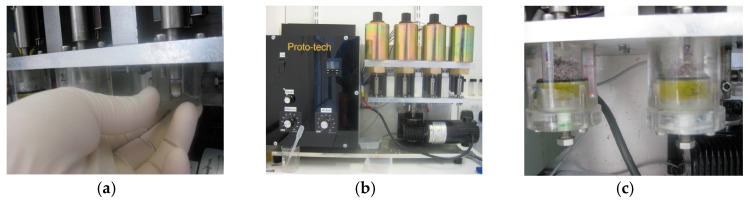
The OHSU oral wear simulator; (**a**) adjustment of the height of antagonist with a custom-made jig and (**b**,**c**) chambers containing the embedded GIC specimen, slurry and antagonist.

**Figure 3:**
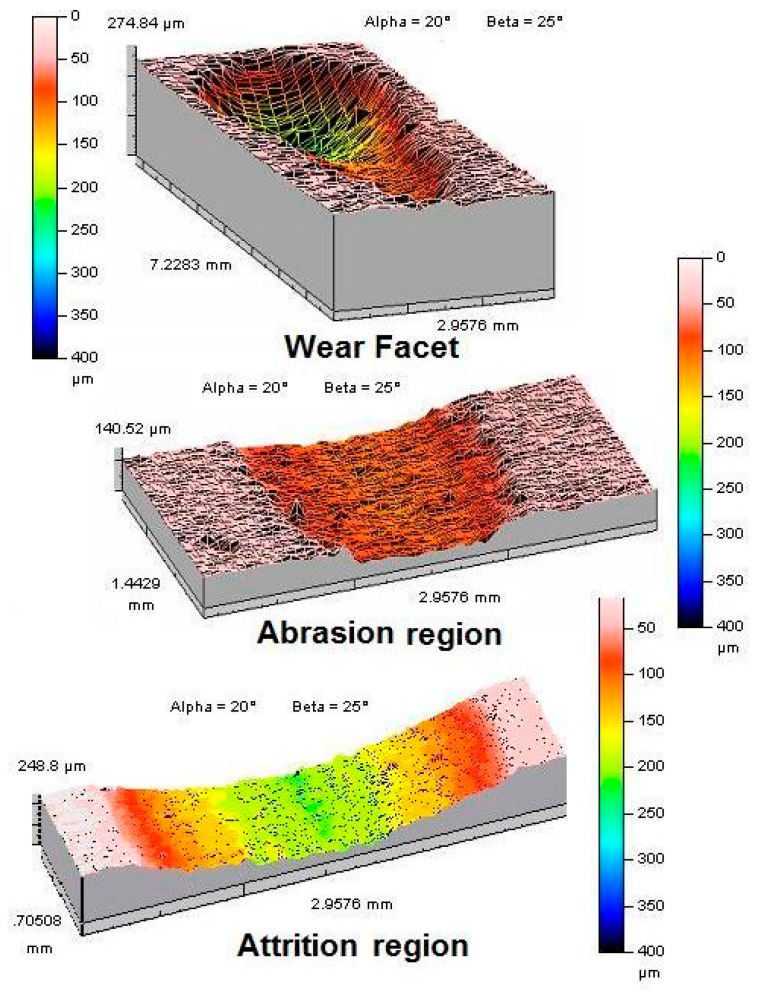
A tear-drop shaped wear facet produced by the OHSU wear simulator showing the abrasion and attrition regions.

The OHSU four-chamber oral wear simulator was used for in vitro wear resistance measurement (Figure 2). The OHSU wear simulator can produce abrasion and attrition wear simultaneously [15]. Ceramic steatite antagonists with a diameter of 5.0 mm (Union Process Inc., Akron, OH, USA) [16] were used. The wear regime of the OHSU oral wear simulator forced the steatite antagonist into contact with the specimen through the food-like slurry and applied a 20 N sliding abrasion force to the surface along a 7-mm linear path. At the end of the 7-mm linear sliding path, a direct static 90 N force was applied to each specimen to simulate attrition wear [15]. The steatite antagonist was raised at the end of each wear cycle and returned to the start of the 7-mm path and the wear regime was repeated for 50,000 wear cycles at a frequency of 1 Hz, which is equivalent to six months wear in the oral environment [15]. The OHSU oral wear simulator produced a tear drop wear facet on the surface of each disc-shaped specimen which contained two regions, abrasion wear and attrition wear. Sliding abrasion wear occurred in the most uniform region of the wear pattern around 40–60% of the 7mm wear trace whereas, during attrition the wear antagonist was stationary and a direct force in the region of 80–90% of the total wear trace length was applied (Figure 3).

Five disc-shaped specimens (12.5 mm of diameter and 2.0 mm of thickness) for each group were prepared using a Teflon mold and stored in deionized water at 37 °C for one hour before they were removed and stored in distilled water at 37 °C for 22 h. The samples were then resin mounted (Varidur; Beuhler, Lake Bluff, IL, USA) to produce cylinders of 25 mm in diameter and 10 mm in height, compatible with the OHSU wear simulator apparatus chambers. The specimens were secured into individual wear chambers of the OHSU oral wear simulator (Figure 2a) after polishing with P600 SiC and P1200 SiC abrasive paper using a Beta grinding-polishing machine (Beuhler, Lake Bluff, IL, USA). The steatite antagonists were fixed to nylon screws (Radionics Ltd., Dublin, Ireland) using a light cured resin-based composite (Grandio; Voco GmbH, Cuxhaven, Germany). The height of the antagonist was adjusted using a custom made jig so that the head of the antagonist was positioned 1 mm above the disc-shaped specimens prior to wear testing (Figure 2b). To simulate three-body wear, a food-like slurry consisted of one gram of poppy seeds (Holland and Barrett, Burton-upon-Trent, UK), 0.5 g of PMMA beads (50–100 μm, Dentsply DeTrey, Kanstanz, Germany) and 5.0 mL distilled water was placed into each wear chamber before testing (Figure 2c).

The tear-drop shaped wear facets were scanned using a noncontact optical profilometer (Talysurf CLI 2000; Taylor Hobson Precision, Leicester, UK) which utilised a 3-mm range chromatic length aberration gauge with a resolution of 0.1 μm (*z*-axis) and scanning speed of 2 mm/s. A series of horizontal traces (perpendicular to the sliding direction of the wear simulator) were conducted across the wear facet at 4 µm intervals (*y*-axis) with longitudinal measurements (parallel to the sliding direction of the wear simulator) taken at 4 µm intervals (*x*-axis) and a detailed three-dimensional representation of the tear drop wear facet was generated (Figure 3) using the TalyMap analysis software package (Taylor Hobson Precision, Leicester, UK).

### 2.4. Vickers Hardness (HV)

A Vickers hardness (HV) test was carried out using a micro-indenter hardness tester (MVK-H1 Mitutoyo, Leeds, UK) with a Vickers diamond indenter in accordance with the ASTM standard C1327-08 [17]. Five disc-shaped specimens (12.5 mm in diameter, 2.0 mm in thickness) for each group were prepared using a Teflon mould. The specimens were stored in deionised water for 24 h. The surface of the specimens was wet-ground with 800 and 1200-grit silicon carbide paper at room temperature prior to testing. A load of 300 N was applied for 10 s and the diamond Vickers indenter created a square impression from which two surface-projected diagonal lengths were measured using an optical microscope (Mitutoyo, Leeds, UK). The mean HV was calculated as the average of nine measurements of each cement groups and three samples were tested for each cement group.

### 2.5. Statistical Analysis

One-way analysis of variance (ANOVA) with the post-hoc Tukey multiple-range test was used to determine the significant differences among the cements in each group for the amount of wear and HV values at associated 95% confidence interval (*p* < 0.05).

## 3. Results

### 3.1. Reciprocating Wear Test

The wear volume and wear depth determined by reciprocating wear test are shown in Table 3. Although the wear rate under the same conditions was different across the wear facets from the area of the first contact to facet centre to the area of last contact, the wear volume values presented here were based on the central deepest part of the wear track to depict the overall wear rate (the value within the brackets represents the standard deviation calculated from five samples). The addition of 1.0 wt % nanoclays in the cement (HiFi-1) resulted in the lowest wear volume 4.90 (0.60) mm^3^ (*p* < 0.05) compared with both HiFi-2 and HiFi-4. However, no significant difference (*p* > 0.05) was found in the wear volume (HiFi, HiFi-2 and HiFi-4) when the data were analyzed by one-way ANOVA and post-hoc Tukey test.

### 3.2. OHSU Wear Test

Figure 3 shows the 3D images of the tear-drop shaped wear facet produced by the OHSU wear simulator. Table 3 shows the comparison of the total wear volume and total wear depth in relation to the mean abrasion and mean attrition values. The total wear volume of HiFi-1, HiFi-2 and HiFi-4 was not significantly different (*p* > 0.05) in the cement groups. The total attrition volume and total abrasion volume of HiFi-2 was significantly different (*p* < 0.05) and the total abrasion volume of HiFi-4 was also statistically different (*p* < 0.05) when compared with the other cements in the same group. The total attrition depth of HiFi was 189 (15.9) µm and was significantly different (*p* < 0.05) when compared with the total attrition wear depth of the other cement groups.

### 3.3. Vickers Hardness (HV)

The results obtained from the HV test are summarized in Table 3. The data were analyzed using a one-way ANOVA and Tukey test and the 95% confidence interval depicted no significant difference (*p* > 0.05) in HV values of all of cements, despite the fact that HiFi-4 (85.4 ± 9) showed the highest values. However, there is no clear indication that incorporation of nanoclays in small amount can improve the HV of cements, when the results were analyzed with a one-way ANOVA and Tukey test.

## 4. Discussion

To date, the assessment of the in vivo or in vitro wear resistance of dental restoratives has been evaluated both in terms of the wear depth and the wear area. Therefore, in order to standardize the wear testing methodologies and data reporting, an appropriate wear quantity namely, area, depth or volume is critical [10]. It should be relevant to the clinical practice and measured easily both in vivo and in vitro using similar or comparable methods [11]. The studies on the wear behavior of materials are based on experiments using simple, unidirectional or reciprocating movements [13,18]. However, the use of different chewing simulation methods for example, the IVOCLAR [19], the Zurich [20], the MTS [21], the ACTA [22], the OHSU [15], the Dento-MunchRobo-Simulator [23] and the University of Alabama [24] wear simulators has been reported. Furthermore, several antagonist materials such as steel [4,25], enamel [4,15,26], polymers (mainly silicon rubber) [27], ceramics (alumina, carbide steel etc.) [28], steatite [29] and titanium [30] have been used to determine wear. It is therefore very difficult to correlate the results of the wear behavior of cements considering the variation in the methodology. The nature of wear is very complex because there are several mechanisms (Table 1 and Table 4)—each of which is sensitive to a wide number of factors that are not necessarily the same or act in the same way [28].

Another important point is that unlike the modulus or strength of a material, the wear testing, does not measure an intrinsic property of the material. Also, there is no a single universal parameter that can be used to characterize the wear behavior of dental cements. The type of the opposing material (antagonist), the loading force and movement patterns are critical factors when wear tests are designed to simulate the oral dental wear. The validation and correlation of the in vitroresults with the in vivo is very complicated because there are no standards for wear characterization, and there is a large number of variables involved in the in vitro vs. the in vivo comparison. International Organization for Standardisation (ISO) published a technical specification ISO 14569-1:1999 (new version: 14569-1:2007) on the “Wear by tooth brushing” [31] followed by ISO 14569-2:2001 “Wear by two- and/or three-body contact” [32]. In the later standard, eight wear-test methods were described, but none were able to reflect the clinical performance [33]. Therefore, significant variations in the methodology of the wear-test parameters such as the type of the wear machine, the antagonist materials, the contact conditions, the applied load, the contact area load and the presence or absence of slurry can be observed in recent studies [19,20,21,22,23,24,25].

The work presented here is aimed toward the development of a process strategy based on the dispersion of nanoclays in PAA aqueous suspensions. The success of the processing strategy, however, relies on the actual dispersion of nanoclays [12,34]. If nanoclays were not penetrated by PAA chains and dispersion was not successful, then agglomeration of nanoclays would have been observed and a micro-composite would have been formed instead of a nanocomposite [34]. Therefore, the size of nanoclays, the nanoclay surface modification method and the processing technique used are important factors for the stability of the PAA-nanoclay suspensions formed. Therefore, the reinforcement of conventional GIC systems with nanoclays at contents more than 2 wt % should be carefully designed because it resulted in an increase in viscosity of the HiFi polymer liquids. It may become necessary to add certain additives such as surfactants to stabilize the nanoclay particles in the PAA solution or using perhaps more dilute PAA solutions. In the present work, a ratio of water: nanoclays = 65:35 was employed. When a PAA-nanoclay suspension was formed, the negative charge of the face/solution interface was due to the isomorphous substitutions of Si and Al atoms in the tetrahedral and octahedral nanoclay sheets, while the charge of the edge/solution interface was determined by the pH of the solution (H^+^ is a crucial ion) [35]. The rheological properties of nanoclays dispersed in an aqueous solution of polyelectrolytes (PAA) were reported by Tejada et al. [36] in order to analyze the changes in the interfacial electric potential of the surface after the dispersion in PAA. They concluded that the addition of nanoclays can provoke dramatic changes in the viscoelastic properties of PAA-nanoclay suspensions depending on the pH, polymer charge and concentration.

**Figure 4:**
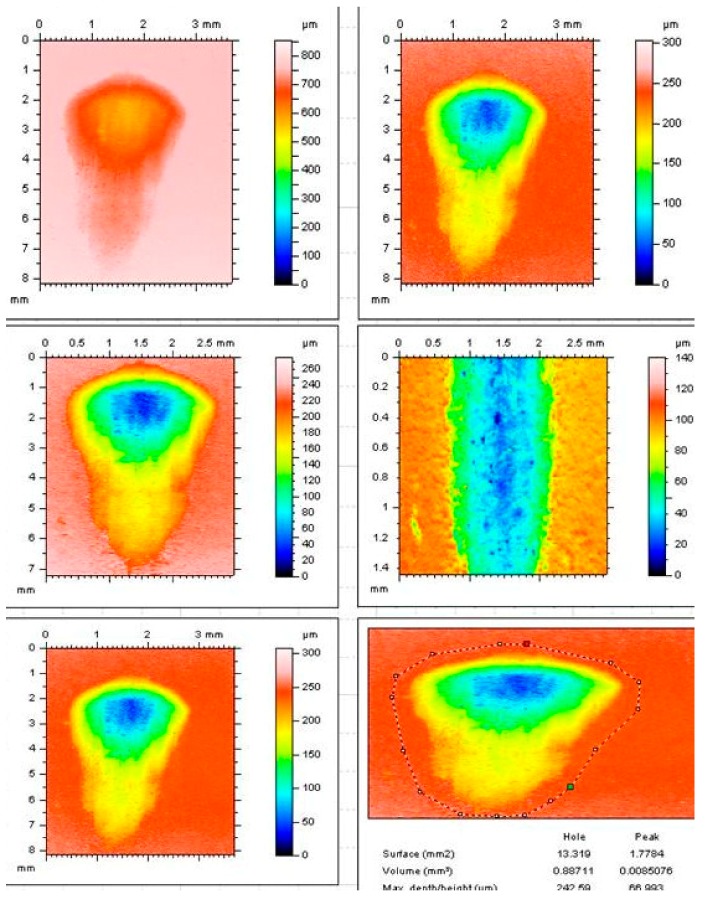
Tear-drop shaped wear facets scanned using a Talysurf optical profilometer (TaylorHobson Precision, Leicester, UK). The wear volume was determined from the wear facet produced by the OHSU wear simulator showing the abrasion region (yellow area, 40–60% of the wear facet length) and the attrition region (blue and green area, 80–90% of the wear facet length).

In the present study, we proposed the dispersion of nanoclays in the liquid portion of GIC to study the wear behavior of the resulting GICs. The reciprocating wear test (ball-on-flat) and the OHSU wear simulation studies were performed in order to investigate the effectiveness of HiFi reinforcement with nanoclays. In the ball-on-flat method, the nature of the wear experiment was simplified to a two-body wear, which may potentially occur as a result of tooth-to-tooth contact without any exogenous third body. In this experiment, however, a three-body wear was also likely to have taken place by worn particles of GIC samples (debris) produced during the testing. In the reciprocating wear test, the cements did not generate wear marks on the alumina antagonist due to the higher hardness of alumina. This suggested that the wear scar was created during the ball-on-flat abrasion and resulted entirely from the wear of the GICs. The results from the OHSU wear simulations, subjected the cement specimens to three wear mechanisms, namely abrasion, three-body abrasion and attrition using a steatite antagonist (Figure 4).

The total wear volume and total wear depth in OHSU wear test were analyzed in terms of their relationship to the mean abrasion and the mean attrition (Figure 3 and Figure 4). The reinforcement of 4 wt % nanoclay (HiFi-4) showed highest total wear volume 1.49 (0.24) mm^3^ in comparison to HiFi-1 0.97 (0.28) mm^3^ and HiFi-2 0.94 (0.21) mm^3^. The abrasion wear resulted in material loss from the GIC specimens, as the steatite antagonist forced through the exposed glass particles when it travelled along the sliding path [37]. In attrition wear, the applied force induced micro cracks that propagated under a cyclic loading, resulting in the removal of material from the GIC surfaces [38]. Heintze et al. analyzed the reproducibility of the mean wear-depth results from different test centres when the OHSU was employed to test three conventional resin composite materials [7]. The difference was 33%–56% at a 20-N abrasion force, whereas the mean wear depth measurements observed for the attrition varied from 31% to 78% at attrition loads from 70 to 90 N.

The calculation of total wear volumes was less dependent upon the abrasion volume and attrition volume. Conversely, the total wear depths were strongly influenced by the attrition depth and to some extent by the abrasion depth. It is therefore suggested that the determination of the total wear volume is more reliable than the total wear depth (Figure 3 and Figure 4). Therefore, the wear regime, from which some of the remaining glass particles were removed from the GIC surface exposing the underlying cement matrix, can be studied in a much better way by using wear volume values. The wear of the steatite antagonist in the OHSU simulation test method may also need to be considered, while measuring the wear resistance. However, the wear of steatite antagonist was not determined in the present study. It would be very useful to monitor the effects of the antagonists’ wear on the wear resistance of GICs.

It was also noted that the OHSU wear simulation produced significantly lower wear values than the ball-on-flat test due to the fundamental differences between these two wear testing methods described in Table 4. A comparison of the various parameters used in these two wear test methods suggested that the wear resistance of GICs obtained from these two different methodologies cannot be correlated and it must be analysed independently.

Considering the factors which may affect the wear of materials, the applied load or pressure, which is defined as the force applied per unit area, may be one of the dominating factors in addition to the type of antagonists used in the determination of wear. One of the factors of fundamental importance, which determines the validation of the in vitro wear determination is the size of the contact area (Table 4). The contact area between the material and the antagonist is inversely proportional to the pressure and consequently can affect the extent and the mechanism of wear in all types of wear studies [8]. In the present work, the ball-on-flat wear test was conducted using alumina ball antagonists whereas, in the OHSU wear simulations steatite antagonists were employed. In both testing methods, the contact area was different resulting therefore in different contact forces. For example, when alumina ball antagonists were employed in the reciprocating wear test method (ball-on-flat), a wear scar on the antagonists was not apparent. Therefore, the smaller contact areas resulted in higher contact forces and therefore more wear of GICs. The larger contact area, in the case of the OHSU simulation method, resulted in smaller contact forces due to the simultaneous wear of the steatite antagonists and the GIC specimens. Krejci et al. evaluated the influence of the size and shape of contact areas on the wear results and found that the wear of restorative materials was inversely related to the contact area size of the antagonistic cusps [39]. They also noted a significant decrease of wear when the contact area increased from 0.26 to 1.18 mm^2^ but surprisingly found no significant difference in the extent of the wear contact areas that increased from 1.18 to 4.10 mm^2^.

Apart from the bulk mechanical properties of cements, the surface properties of dental cements are as important, especially when considering that these materials function in the oral environment. The results obtained from the Vickers hardness (HV) test showed no significant differences (*p*> 0.01) in the HV values of all cements. The average values of hardness were obtained between 68 and 85 HV and the presence of nanoclays did not influence the hardness of glass ionomer cements.

## 5. Conclusions

The total wear depths were strongly influenced by the attrition depth, and to some extent by the abrasion depth, and it is suggested that the determination of the total wear volume is more reliable than the total wear depth. This argument is strongly supported by the wear tests conducted by both the reciprocating wear test method and the OHSU wear simulation method. The contact area between the material and the antagonist is inversely proportional to the load and can affect the extent and the mechanism of wear in wear studies. The total wear volume of the HiFi cements after the dispersion of nanoclays (4 wt %) generally increased compared to the control. The HV of cements was calculated between 62 and 89 HV and no significant difference was observed between the GICs after the addition of nanoclays when the data were statistically analyzed.

## Figures and Tables

**Table 1:** List of factors affecting wear resistance of materials (modified from [8]).

**Material Parameters**	Composition, microstructure, mechanical properties (modulus, yield strength, ductility), fracture toughness, hardness, Poisson’s ratio
**Design Parameters**	Shape and type of antagonist, loading, force/impact level, type of motion, roughness, cycle time
**Environmental Parameters**	Temperature, humidity, atmosphere, wet or dry condition, pH, contamination and so on
**Lubrication Parameters**	Type of medium, presence of slurry, stability of slurry

**Table 2:** Schematic presentation of polymer-nanoclay liquid formation and subsequent formation of glass ionomer cements.

**GIC Liquid**	**Nanoclays (wt %)**	**Water (%)**	**PAA Powder (%)**
PAA	0.0	60	40
PAA1	1.0	59	40
PAA2	2.0	58	40
PAA4	4.0	56	40
**Cement Specimen**	**GIC Liquid**	**Powder**	**P/L Ratio**
HiFi	PAA	HiFi glass	4.2:1
HiFi-1	PAA1	HiFi glass	4.2:1
HiFi-2	PAA2	HiFi glass	4.2:1
HiFi-4	PAA4	HiFi glass	4.2:1

**Table 3:** Hardness and wear data of HiFi cements reinforced with nanoclays (*p* < 0.05).

Test Methods	Parameters	HiFi	HiFi-1	HiFi-2	HiFi-4
Reciprocating wear test	Volume (mm^3^)	6.08 (2.1) ^a^	4.90 (0.6) ^b^	5.66 (1.0) ^a^	6.22 (1.3) ^a^
	Depth (µm)	481 (30) ^a^	419 (43) ^b^	512 (117) ^a^	419 (63) ^b^
OHSU Volumetric Wear (mm^3^)	Total wear volume	0.69 (0.13) ^a^	0.95 (0.22) ^b^	0.97 (0.28) ^b^	1.49 (0.24) ^b^
	Attrition volume	0.15 (0.03) ^a^	0.28 (0.03) ^b^	0.21 (0.05) ^c^	0.31 (0.07) ^b^
	Abrasion volume	0.07 (0.01) ^a^	0.14 (0.01) ^b^	0.09 (0.03) ^a^	0.24 (0.04) ^c^
OHSU Wear Depth (µm)	Total wear depth	222 (17) ^a^	291 (35) ^b^	291 (33) ^b^	343 (44) ^b^
	Attrition depth	189 (15.9) ^a^	245 (24) ^b^	247 (33) ^b^	300 (37) ^c^
	Abrasion depth	92.40 (5.5) ^a^	147 (20) ^b^	111 (18) ^b^	165 (23) ^b^
Vickers Hardness (HV)		72.50 (3.2) ^a^	62.70 (6.6) ^a^	69.20 (4.1) ^a^	85.40 (9.9) ^a^

^a,b,c^ The groups annotated with different superscript lower case letters in a row indicate statistically significant differences.

**Table 4:** A comparison of the various parameters used in reciprocating wear test and the OHSU wear test.

Parameters		Reciprocating Wear Test	OHSU Wear Test
Mechanism		Ball-on-flat	Simulation
Type		Two-body wear	Three-body wear
Force (N)		20	20 and 90
Sliding distance (mm)		6	7
Contact duration		All time (to-and fro)	Plough
Antagonist	Material	Alumina (Al_2_O_3_)	Steatite magnesium silicate
	Diameter (mm)	12.5	5.0
	Hardness (HV)	1700	650
	Tendency to wear	Yes	No
No. of cycles	Total cycles	10,000	50,000
	Total time (h)	4	12
	Frequency (Hz)	1	1
Medium	Distilled water	Yes	Yes
	Slurry	No	Yes
	Temperature	Room temp.	Room temp.
Profilometer	Type	Contact stylus	Non-contact optical
	Number of scans	3	1750
	Step size	1 mm	0.004 mm
